# A Rationally Designed Humanized Antibody Selective for Amyloid Beta Oligomers in Alzheimer’s Disease

**DOI:** 10.1038/s41598-019-46306-5

**Published:** 2019-07-08

**Authors:** Ebrima Gibbs, Judith M. Silverman, Beibei Zhao, Xubiao Peng, Jing Wang, Cheryl L. Wellington, Ian R. Mackenzie, Steven S. Plotkin, Johanne M. Kaplan, Neil R. Cashman

**Affiliations:** 1 University of British Columbia, Djavad Mowafaghian Centre for Brain Health, Vancouver, BC V6T 2B5 Canada; 2 University of British Columbia, Department of Physics and Astronomy, Vancouver, BC V6T 1Z1 Canada; 3 University of British Columbia, Department of Physics and Astronomy and Genome Sciences and Technology Program, Vancouver, BC V6T 1Z1 Canada; 4 ProMIS Neurosciences, Cambridge, MA 02142 USA

**Keywords:** Peptide vaccines, Alzheimer's disease

## Abstract

Advances in the understanding of Alzheimer’s disease (AD) suggest that pathogenesis is not directly related to plaque burden, but rather to soluble toxic amyloid-beta oligomers (AßO). Therapeutic antibodies targeting Aß monomers and/or plaque have shown limited efficacy and dose-limiting adverse events in clinical trials. These findings suggest that antibodies capable of selectively neutralizing toxic AßO may achieve improved efficacy and safety. To this end, we generated monoclonal antibodies against a conformational Aß epitope predicted by computational modeling to be presented on toxic AßO but not monomers or fibrils. The resulting lead antibody, PMN310, showed the desired AßO-selective binding profile. *In vitro*, PMN310 inhibited AßO propagation and toxicity. *In vivo*, PMN310 prevented AßO-induced loss of memory formation and reduced synaptic loss and inflammation. A humanized version (huPMN310) compared favorably to other Aß-directed antibodies showing a lack of adverse event-associated binding to Aß deposits in AD brains, and greater selective binding to AßO-enriched AD brain fractions that contain synaptotoxic Aß species. Systemic administration of huPMN310 in mice resulted in brain exposure and kinetics comparable to those of other therapeutic human monoclonal antibodies. Greater selectivity for AßO and the potential to safely administer high doses of huPMN310 are expected to result in enhanced safety and therapeutic potency.

## Introduction

Strong genetic and experimental evidence supports a causative role for amyloid-beta (Aß) in the pathogenesis of Alzheimer’s disease (AD)^1–4^. However, views on the nature of the Aß species responsible for progressive neurodegeneration have evolved with greater understanding of AD pathogenesis. Aß plaque is a hallmark of AD and was initially believed to be responsible for neuronal cell death. However, a mounting body of experimental and clinical data has shown that soluble toxic Aß oligomers (AßO), rather than insoluble fibrils and plaque, appear to be the primary drivers of synaptic dysfunction, neuronal loss and cognitive decline in AD patients^5,6^. Plaque burden correlates poorly with memory impairment^7,8^ and insoluble Aß fibrils show little or no demonstrable toxicity *in vitro* or *in vivo*^9,10^. In contrast, a significant correlation exists between disease severity and levels of soluble Aß in the central nervous system^11^, commensurate with the high degree of neurotoxicity and induction of cognitive impairment exhibited by soluble AßO *in vitro* and *in vivo*^7–10,12^.

This current understanding of Aß toxicity, as dominated by AßO, may explain the observed clinical failure of antibodies targeting other forms of Aß without directly or effectively impacting toxic oligomers (reviewed in^13^). For example, solanezumab, designed to bind Aß monomers, was found to be safe but lacked efficacy in large Phase III clinical trials^14^. Similarly, small molecule inhibitors of ß-amyloid cleaving enzyme (BACE), aimed at preventing the generation of monomers, have also failed to show efficacy in pivotal trials^15^. Even though this approach might be expected to reduce oligomer formation, the degree of inhibition may be insufficient and does not address the pre-formed reservoirs of oligomers that exist in equilibrium with large aggregates and plaque^16^.

Other antibodies, which bind all forms of Aß (e.g. bapineuzumab), were also found to lack efficacy^17^, in line with the likelihood that competition binding to abundant non-toxic Aß monomers and plaque would result in a considerable reduction of the effective dose against toxic AßO. In addition, antibody binding to Aß plaque and vascular Aß deposits has been associated with an increased risk of amyloid-related imaging abnormalities (ARIA), namely brain edema (ARIA-E) and micro-hemorrhages (ARIA-H), in particular for antibodies with an IgG1 effector isotype^18,19^. Antibodies such as aducanumab and BAN2401 actively bind AßO without unproductive binding to Aß monomers^20,21^. However, their binding to Aß fibrils has resulted in the occurrence of dose-limiting ARIA-E^20,21^. No clinical stage antibodies tested thus far display selectivity for toxic AßO, while avoiding binding to Aß monomers or plaque.

The weight of available evidence indicates that antibodies selective for the toxic Aß oligomer species are needed in order to achieve optimal efficacy and safety in the treatment of AD. To address this challenging requirement, we generated monoclonal antibodies against a conformational epitope predicted by computational modeling to be presented by toxic AßO, but not by monomers or fibrils. The resulting lead antibody candidate, PMN310, selectively targeted and neutralized AßO with no significant reactivity to Aß monomers or fibrils.

## Results

### Selectivity of monoclonal antibody PMN310 for Aβ oligomers

**Figure 1:**
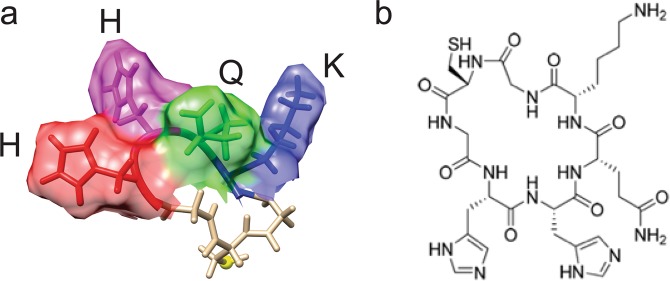
Aβ residues 13–16, HHQK, held in a constrained turn represent a predicted AβO-specific epitope. (**a**) Representative molecular dynamics simulated conformation of the cyclic HHQK epitope, with the side chains oriented into solvent. Yellow dot is sulfhydryl on terminal cysteine, used for conjugation to carrier proteins. (**b**) Skeletal structural formula of c[CGHHQKG].

Mouse monoclonal antibodies selective for AβO were generated by immunization with a conformational peptide epitope predicted to be exposed on misfolded toxic oligomers but not on monomers or insoluble fibrils. Computational modeling using collective coordinates^22^ was used to identify regions and structures of Aβ thermodynamically likely to be exposed in oligomers. The candidate epitope, amino acid sequence HHQK (Aβ residues 13–16), in a predicted AβO-specific conformation, was replicated in the form of a cyclic peptide, c[CGHHQKG], depicted in Fig. 1, which was then used to immunize mice. Monoclonal antibody clones raised against c[CGHHQKG] were screened by ELISA for binding to the structured c[CGHHQKG] conformational epitope, with little or no binding to the unstructured linear form of the peptide CGHHQKG (Supplementary Fig. 1).

**Figure 2:**
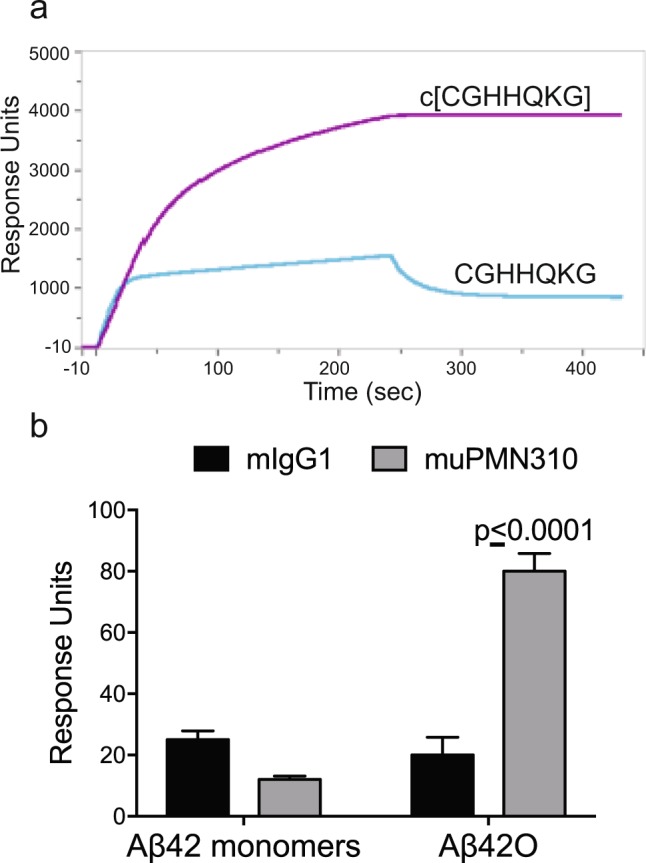
Selective binding of the muPMN310 antibody to cyclized CGHHQKG peptide and Aβ oligomers. (**a**) SPR measurements of muPMN310 binding to cognate cyclized peptide epitope c[CGHHQKG], and unstructured linear CGHHQKG peptide. Representative sensorgrams from two independent experiments are shown. (**b**) SPR measurements of muPMN310 binding to Aβ42 monomers and oligomers (Aβ42O). Means and SEM of three identical experiments are shown. Differences were determined by two-way ANOVA with Sidak’s multiple comparison test: muPMN310 binding to Aβ42O is significantly higher than all other measurements (p ≤ 0.0001); muPMN310 binding to Aβ42 monomers is not different from isotype control (mIgG1) by two-way ANOVA.

Selected antibody clones underwent further testing, results for the lead murine antibody clone, muPMN310, are shown in Fig. 2. Surface plasmon resonance (SPR) showed preferential binding to the structured cyclic form of the peptide, predicted to be specifically exposed on AβO, versus the unstructured linear peptide epitope present on Aβ monomers (Fig. 2a; kinetic analysis of binding in Supplementary Fig. 2). AβO selectivity was confirmed by SPR showing robust binding of muPMN310 to synthetic Aβ42O over monomers (Fig. 2b).

**Figure 3:**
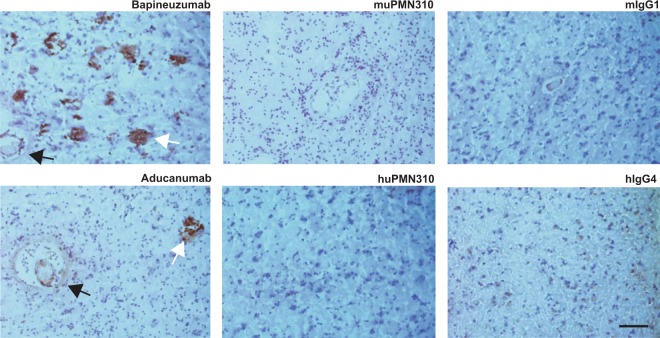
PMN310 does not react with Aβ plaque or vascular deposits in AD brain sections. Sections from the frontal cortex of human AD brain were stained with 1 μg/ml of indicated antibodies. Detection of bound antibody with secondary anti-human or anti-mouse IgG appears in brown, and nuclear counterstaining with hematoxylin in blue. The images are representative of 3 or more independent experiments with four individual AD brains. Scale bar = 100 μm, applicable to all images. White arrows - characteristic plaque staining; black arrows - Aβ vascular deposit staining.

Importantly, muPMN310 failed to bind Aβ fibrillar plaque in frozen brain sections of frontal cortex from confirmed AD patients, in contrast to other Aβ-directed antibodies which produced robust immunoreactivity (Fig. 3). The lack of muPMN310 binding to plaque was not due to a loss of the conformational epitope under the staining conditions used since microscopy slides coated with a sample of the BSA-conjugated conformational cyclic peptide epitope submitted to the same staining protocol as the brain tissue sections produced clear staining by muPMN310 and not a mouse IgG control (Supplementary Fig. 3). Together the results demonstrate that PMN310 binds AβO, with no reactivity to Aβ monomers or fibrils.

### Inhibition of AβO biological activity by PMN310

**Figure 4:**
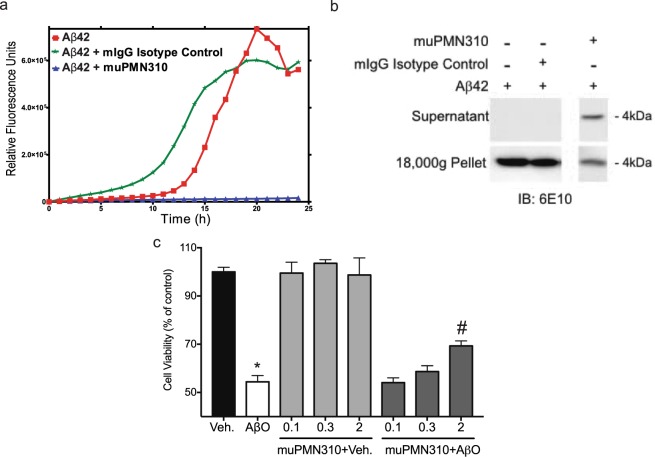
muPMN310 binding inhibits Aβ42 aggregation and Aβ42O toxicity *in vitro*. (**a**) β-sheet formation was tracked for 24 hours *in vitro* with a Thioflavin T fluorescent assay after addition of Aβ monomers alone (red line), or in the presence of muPMN310 (blue line) or isotype control mIgG (green line) at a molar ratio of 1:5 (Antibody:Aβ). Data are representative of three independent experiments. (**b**) Thioflavin T fluorescent assay samples were collected at end-point and fractionated into soluble Aβ (Supernatant; monomers, small oligomers) or insoluble Aβ (Pellet; large aggregates) by centrifugation, then run on a denaturing SDS-PAGE gel for monomerization and immunoblotting with pan-Aβ antibody, 6E10. Shown are representative data from one of two identical experiments. (**c**) MTT assay of viability of neurons treated with vehicle (Veh.) or AβO and increasing molar ratios of muPMN310. Antibody:AβO ratios of 1:10 (0.1), 1:3 (0.3), 2:1 (2). Mean ± SEM shown. Differences were determined with a Students t-test: (*) Veh. vs AβO p ≤ 0.0001, (#) AβO vs muPMN310 + AβO p = 0.0112. Similar results were observed in 3 independent assays.

*In vitro* assays were conducted to determine whether binding of muPMN310 translated into biological activity against AβO. The ability of muPMN310 to interfere with propagation of Aβ42 aggregation was tested in a Thioflavin-T fluorescence β-sheet formation assay. Aβ42 peptide incubated alone showed a steady increase in fluorescence until a plateau was reached after approximately 24 h (Fig. 4a). The addition of muPMN310 at an antibody:Aβ42 molar ratio of 1:5 (an excess molar ratio of Aβ to assess the blocking potential of PMN310) completely inhibited accumulation of β-sheet structure (Fig. 4a). Conversely, control mouse IgG had no blocking effect (Fig. 4a).

To validate this finding, we collected samples at the endpoint of the Thioflavin-T assay, and separated insoluble aggregates from soluble monomers and oligomers by centrifugation. The supernatant (containing soluble monomeric and small oligomeric Aβ), and pellets (containing insoluble Aβ fibrils and aggregates), were subjected to denaturing/disaggregating SDS-PAGE, to monomerize Aβ, followed by immunoblotting with a pan-Aβ antibody. As a result of the denaturing SDS-PAGE, the Aβ band at 4 kDa represents a majority of Aβ in the original samples. The immunoblots show that addition of muPMN310 to Aβ42 peptides maintained a pool of soluble Aβ in the supernatant at assay endpoint, whereas no soluble Aβ remained for Aβ42 incubated alone or with an isotype control (Fig. 4b). Correspondingly, strong Aβ signals were observed in the pelleted insoluble fractions from Aβ42 incubated alone or with isotype control, while a reduced insoluble Aβ signal was obtained with the Aβ42 + muPMN310 sample (Fig. 4b, uncropped immunoblots provided in Supplementary Fig. 4a). Together these results suggest that muPMN310 inhibits aggregation of Aβ42.

In separate assays, the ability of muPMN310 to counteract the toxicity of synthetic AβO (Supplementary Fig. 4b,c) was tested in cultures of primary mouse cortical neurons to provide a proof of concept assessment of its neuroprotective potential. As shown in Fig. 4c, the presence of AβO significantly diminished the metabolic activity of neurons as assessed in an MTT colorimetric assay at 24 h (56% viability vs vehicle control). The addition of muPMN310 to the cultures at antibody:AβO molar ratios of 0.1–2 resulted in dose-dependent inhibition of oligomer toxicity with statistical significance at the highest ratio. The addition of muPMN310 alone to neuronal cell cultures as a control had no effect on viability (Fig. 4c).

**Figure 5:**
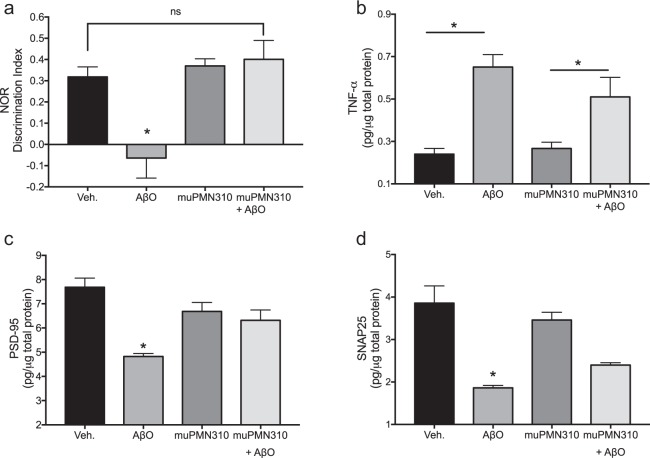
muPMN310 inhibits AβO toxicity *in vivo*. Wild-type mice (n = 12 per group) were injected i.c.v. with either vehicle (Veh.), Aβ42O + vehicle (AβO), muPMN310 + vehicle (muPMN310) or muPMN310 + AβO at a molar ratio of 2:1. (**a**) NOR discrimination indices (n = 7–10 evaluable mice per group). Mean ± SEM shown. Data were not normally distributed. Differences were compared using Kruskal-Wallis ANOVA with Dunn’s multiple comparison test. AβO is significantly less than all other samples (*p ≤ 0.05 vs Vehicle, muPMN310, muPMN310 + AβO). No other comparisons were significant, including Veh.:PMN310 + AβO. Hippocampal levels of TNF-alpha (**b**), PSD-95 (**c**), SNAP25 (d) (n = 11–12 mice per group). Mean ± SEM shown. Differences were compared using one-way ANOVA with Tukey’s multiple comparison test for normally distributed data in (**b**: *p ≤ 0.05) and (**c**). (**d**) Difference determined with Kruskal-Wallis ANOVA with Dunn’s multiple comparison test for non-normally distributed data. In (**c**,**d**) *p ≤ 0.05 for AβO vs Veh., muPMN310, muPMN310 + AβO.

The ability of muPMN310 to inhibit oligomer neurotoxicity was also assessed *in vivo* in WT mice injected intracerebroventricularly (i.c.v.) with synthetic AβO (Supplementary Fig. 4) with or without muPMN310. Mice injected with AβO alone displayed a profound deficit in short-term memory formation as assessed one week later in a novel object recognition (NOR) test (Fig. 5a). AβO-injected mice failed to recognize a new object and displayed a discrimination index of 0 or less. Co-injection of muPMN310 with the toxic oligomers completely prevented this cognitive deficit and the treated mice displayed a discrimination index no different from that of control mice. As expected, i.c.v. injection of muPMN310 alone had no effect (Fig. 5a).

The cognitive deficit induced by i.c.v. injection of AβO was associated with inflammation and synaptic damage in the hippocampus, a region important in the development of memory. Hippocampal homogenates from AβO-treated mice displayed an increase in levels of TNF-alpha (pro-inflammatory marker; Fig. 5b) and decreases in PSD-95 (postsynaptic marker; Fig. 5c) and SNAP25 (presynaptic marker; Fig. 5d) by ELISA. Partial protection from these changes was observed in mice co-injected with synthetic AβO and muPMN310. Nevertheless, the observed partial inhibition was sufficient to fully restore short-term memory formation in these animals (Fig. 5a). This observation is in line with the notion that some level of neuronal impairment is tolerable (or can be compensated for), such that AD patients can function normally and remain asymptomatic for many years in spite of progressive neuronal damage. Finally, i.c.v. injection of muPMN310 alone as a control, had no effect on the molecular markers measured (Fig. 5b–d).

Overall, these results indicate that the muPMN310 antibody generated by immunization with a computationally predicted AβO-restricted epitope, shows selective binding for AβO over Aβ monomers or plaque and inhibits the activity of AβO *in vitro* and *in vivo*.

### Comparison of humanized PMN310 binding profile to other Aβ-directed antibodies

In view of the favorable properties of muPMN310, the antibody was humanized for therapeutic application. An IgG4 isotype was specifically chosen for its low effector function and consequent ability to optimally achieve neutralization and clearance of AβO without causing inflammation. The humanized antibody (huPMN310) retained selective binding for synthetic AβO vs Aβ monomers, as assessed by SPR (Supplementary Fig. 5). Lack of plaque binding with huPMN310 was also confirmed by immunohistochemistry on frozen frontal cortex brain sections from AD patients. As shown in Fig. 3, the Aβ-directed antibodies bapineuzumab and aducanumab, known to bind fibrils, showed robust staining of parenchymal Aβ plaque and vascular deposits while neither muPMN310 nor huPMN310 showed any staining above background. Similar results were obtained with brain sections from aged APP/PS1 transgenic mice (Supplementary Fig. 6). The selectivity of huPMN310 for soluble toxic oligomers with no binding to plaque or Aβ monomers is a therapeutic advantage as it is expected to prevent the loss of antibody to unproductive interactions and, more importantly, avert triggering dose-limiting induction of ARIA-E and ARIA-H.

### Binding of huPMN310 to soluble AD brain extracts

**Figure 6:**
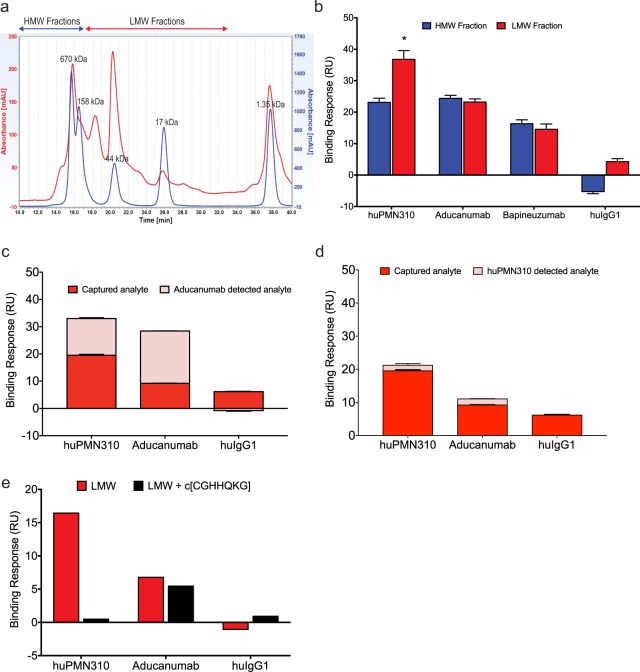
huPMN310 selectively recognizes soluble, low molecular weight, Aβ aggregate species in AD brains. (**a**) SEC fractionation chromatogram of pooled soluble AD human brain extracts. A representative chromatogram from 9 independent fractionations is shown (red line). MW markers are superimposed for reference (blue line). (**b**) SPR binding response of indicated antibodies to pooled LMW and HMW fractions. Four measurements were made under identical conditions: statistical significance of differences was determined by two-way ANOVA with Sidak’s multiple comparison test: LMW binding by huPMN310 was greater than all other measurements (p < 0.0001), huIgG1 (LMW and HMW) binding was significantly less than all other measurements (p ≤ 0.0012). No significant difference between LMW and HMW binding for aducanumab vs bapineuzumab. (**c**,**d**) SPR binding response of indicated antibodies to LMW AD brain extract and subsequent aducanumab (**c**) or huPMN310 (**d**) detection of the analyte captured. Fifteen capture and three detection measurements under identical conditions were performed. (**e**) c[CGHHQKG] peptide was pre-injected over indicated immobilized antibody surfaces to compete with LMW brain analyte. Data are representative of two experiments with similar results.

It is recognized that synthetic AβO, while a useful tool, do not exactly replicate the structural plasticity, conformational heterogeneity, co-aggregation species and polydispersion of native AβO found in AD brains. Examination of soluble Aβ species in AD brain extracts by several investigators has indicated that the neurotoxic activity resides primarily in the low molecular weight (LMW) fraction of AβO (dimers, trimers, tetramers, dodecamers), while high molecular weight (HMW) aggregates are largely inert, though they reportedly can dissociate into LMW species^7,8,10,12,16,23^. Therefore, size exclusion chromatography (SEC) of pooled soluble extracts from AD brains was performed to confirm the binding of huPMN310 to native AβO and, in particular, the LMW species. SEC fractionation of soluble AD brain extract gave rise to a highly reproducible pattern (Fig. 6a). Fractions corresponding to ~8–70 kDa were pooled into a LMW fraction, expected to contain AβO in the dimer to dodecamer range, and excluding monomers. Fractions corresponding to >140–700 kDa were pooled into a HMW fraction. Measurements of total Aβ38, 40 and 42 by MESO scale analysis showed all three species to be present in both the LMW and HMW fractions with Aβ42 representing the major aggregated (oligomeric) Aβ species (Table 1).

**Table 1:** AβOs are present in LMW and HMW fractions of AD brain extracts.

Aβ species	LMW fraction (pg)	HMW fraction (pg)
Aβ42 Aggregated	1.3	43.8
Aβ42 Total	118	92
Aβ40 Aggregated	0.37	3.4
Aβ40 Total	1311	1305
Aβ38 Aggregated	Below detection	Below detection
Aβ38 Total	958	953

LMW and HMW fractions from soluble extracts of 3 pooled AD brains were analyzed for amounts of total (MSD platform assay) and aggregated (A4 assay) Aβ42, Aβ40 and Aβ38 species.

Binding of immobilized huPMN310 and other Aβ-directed antibodies to the LMW and HMW fractions of pooled soluble AD brain extract was assessed by SPR (Fig. 6b; sensorgrams and immunoblot presented in Supplementary Fig. 7). The huPMN310 antibody consistently showed high and preferential binding to the LMW fraction. The same binding pattern was observed with individual AD brains or pooled extract (Supplementary Fig. 8A). In contrast, aducanumab and bapineuzumab showed lower and non-preferential binding to the LMW vs HMW fraction. With pooled non-AD control brain extract (Supplementary Fig. 8B), aducanumab showed greater binding to the HMW fraction and binding to the LMW fraction comparable to that of huPMN310. This is consistent with the selectivity of aducanumab for any form of aggregated Aß and the preferential recognition of small Aß oligomers by huPMN310.

Aβ protein and Aβ aggregates were determined to be present in the AD LMW fraction (Table 1), but represent only a subset of total proteins in the brain extract. To confirm that huPMN310 was in fact binding to Aβ in the LMW fraction, a sandwich SPR assay was conducted whereby the material captured by immobilized huPMN310 was subsequently exposed to a detector antibody. Aducanumab was chosen as the detector antibody as it is known to recognize aggregated Aβ, and was expected to bind/detect material captured by immobilized aducanumab, providing a positive control. Aducanumab also typically produced a greater binding response to brain extract than bapineuzumab, thereby enhancing the sensitivity of SPR measurements. As demonstrated in Fig. 6c, aducanumab detection in the sandwich assay produced a robust binding response to material captured by either huPMN310 or aducanumab, thereby confirming the binding of huPMN310 to AβO in the LMW fraction (sensorgram curves are provided in Supplementary Fig. 9). The material giving rise to the low signal observed after capture with control human IgG was not detected by aducanumab, consistent with a low degree of non-specific background binding.

Using huPMN310 as the detector antibody in the same assay, we observed detection of PMN310- and aducanumab-captured material (Fig. 6d). The signal is reduced compared to aducanumab detection (Fig. 6c), consistent with binding sites on LMW AβO being largely occupied by the PMN310 capture antibody. There was also lower detection of aducanumab-captured material, possibly because aducanumab largely captures Aβ assemblies different from those recognized by PMN310. As expected, there was no detection of the material bound non-specifically by control human IgG1.

The specificity of huPMN310 binding was further demonstrated in a competition assay. Pre-exposure of immobilized huPMN310 to its cognate cyclic peptide epitope, c[CGHHQKG], completely prevented subsequent binding to the LMW fraction of AD brain extract (Fig. 6e). By comparison, pre-exposure of immobilized aducanumab to c[CGHHQKG] had no appreciable impact on subsequent binding to the LMW fraction since aducanumab is specific for a different epitope (Aβ residues 3–7) and does not bind c[CGHHQKG] (Supplementary Fig. 2)^24^. Taken together, these results suggest that huPMN310, in addition to being selective for AβO vs monomers and plaque, also exhibits superior targeting of the LMW toxic oligomer-enriched fraction of AD brain extract compared to other Aβ-directed antibodies.

### CNS penetrance of huPMN310

**Figure 7:**
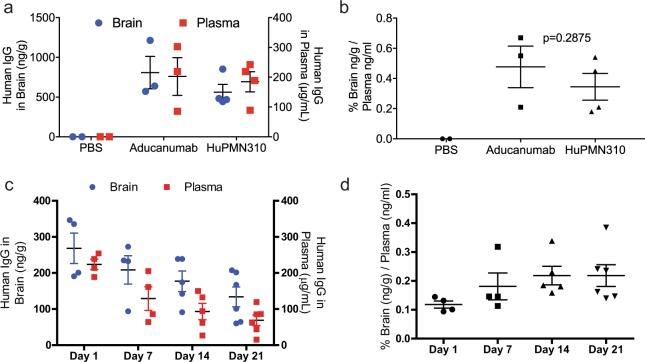
CNS penetrance of huPMN310. (**a**,**b**) Aged WT C57Bl/6 mice injected i.p. with 30 mg/kg of aducanumab (n = 3), huPMN310 (n = 4) or PBS (n = 2). (**a**) Plasma and brain levels of human IgG at 24 h. No statistically significant difference between aducanumab and huPMN310 in plasma or brain by two-way ANOVA. (**b**) CNS penetrance - Percent of human IgG in brain compared to plasma. No statistically significant difference between aducanumab and huPMN310 by Mann-Whitney test. (**c**,**d**) Aged APP/PS1 mice injected i.p. with 30 mg/kg of huPMN310. (**c**) Plasma and brain levels of huPMN310 at days 1–21 (n = 4–6 mice/time point). Two-way ANOVA with Sidak’s post-test test shows statistically significant differences in plasma levels between day 1 and days 7 (p = 0.0016), 14 and 21 (p < 0.0001) with no significant differences in brain levels at the different time points. (**d**) Brain:plasma ratios of huPMN310 at days 1–21. Kruskal-Wallis ANOVA with Dunn’s post- test shows a statistically significant difference only for Day 1 vs 14 (p = 0.043).

In order to achieve clinical efficacy, therapeutic levels of huPMN310 need to be delivered to the CNS. Therefore, the ability of huPMN310 to cross the blood brain barrier (BBB) and enter the CNS from the periphery was assessed and compared to that of aducanumab in aged WT mice (15–17 months old). Mice were dosed with a single intraperitoneal (i.p.) injection of 30 mg/kg antibody and levels of human IgG present in the plasma and perfused brains were measured 24 h later by ELISA. As shown in Fig. 7, equivalent amounts of huPMN310 and aducanumab were detected in plasma and brain (Fig. 7a) demonstrating a comparable degree of CNS penetrance (Fig. 7b) in the range of ~0.3% as previously reported for aducanumab^20^. As expected, no human IgG was detected in mice injected with PBS alone as a negative control (Fig. 7a).

Additionally, a time course study was conducted in aged (13–17 months old) transgenic APP/PS1 mice in order to assess the pharmacokinetics of huPMN310. Plasma and brain levels of human IgG were measured on days 1–21 after i.p. administration of 30 mg/kg huPMN310. In spite of declining plasma levels, CNS levels of huPMN310 were detectable out to the study endpoint at day 21 (Fig. 7b,c). Interestingly, APP/PS1 mice showed a trend (albeit not statistically significant) for greater CNS retention of huPMN310 over time compared to age-matched WT mice, consistent with engagement of target AβO present in the brain of transgenic mice but not WT littermates (Supplementary Fig. 10). Taken together, these results suggest that the CNS penetrance of huPMN310 is comparable to that of other monoclonal antibodies against Aβ targets^14,20,25^.

## Discussion

In the current study, an antibody with greater selectivity for toxic AβO was generated to maximize efficacy against AD by reducing unproductive binding to the more abundant non-toxic Aβ species (monomers, large/HMW soluble aggregates, insoluble fibrils/plaques) and increase safety by preserving normal Aβ function and decreasing the risk of adverse effects (ARIA) associated with the engagement of plaque and vascular deposits^18–20^. Aβ oligomers consist of a heterogeneous mixture of aggregates which necessarily share epitopes with monomers and fibrils. As such, traditional immunization with AβO is unlikely to yield toxic oligomer-specific antibodies without cross-reactivity to monomers or fibrils. A computational modeling approach was therefore used to predict conformational epitopes thermodynamically likely to be exposed on misfolded toxic oligomers only^22,26,27^. Monoclonal antibodies were raised against the putative conformational epitope c[CGHHQKG] and were validated empirically for selective binding and ability to neutralize toxic oligomers. This approach successfully led to the generation of lead antibody muPMN310 with the desired binding profile and activity.

The selectivity of muPMN310 for AβO was confirmed by SPR showing preferential binding to synthetic oligomers over monomers and by the lack of binding to Aβ plaque and vascular deposits in AD brain sections. In contrast, other clinical-stage Aβ-directed antibodies also bind monomers and/or fibrils in addition to AβO (Fig. 3)^13,20,28–30^. *In vitro*, muPMN310 prevented propagation of Aβ42 aggregation and inhibited the neurotoxicity of synthetic oligomers in primary neuronal cultures (Fig. 4a–c). *In vivo*, muPMN310 blocked the AβO-induced loss of short-term memory formation (NOR assay) and the observed cognitive benefit was associated with an improvement in hippocampal markers of synaptic loss and inflammation (Fig. 5a–d).

Based on these properties, the muPN310 antibody was humanized for clinical application. Most clinically studied antibodies to date possess an IgG1 isotype with effector function^13^. The IgG1 isotype offers the highest degree of interaction with Fc-gamma receptors on microglial cells thereby promoting the removal of antigen-antibody complexes via phagocytosis, but also causing activation and release of pro-inflammatory cytokines in the process^20,28,29,31^. By comparison, the IgG4 isotype shows weaker Fc-gamma receptor interaction resulting in reduced uptake of antigen-antibody complexes by microglial cells but without causing activation. For example, in a side-by-side comparison of human IgG1 and IgG4 versions of the same Aß-specific antibody, Adolfsson *et al*. showed that while the IgG4 version induced lower uptake of AßO by microglial cells *in vitro*, it did so without triggering a pro-inflammatory response^29^. Several lines of evidence suggest that Fc effector function is not a requirement to achieve Aß clearance *in vivo* such as activity of Fab fragments or efficacy of antibodies in APP Tg2576 Fc receptor gamma knock-out mice^32^.

Notably, clearance of antigen-antibody complexes from the brain does not occur solely through phagocytosis but via several other mechanisms including non-specific, isotype-independent CSF glymphatic flow^33^ and transport into the circulation via the FcRN receptor which interacts equally well with all IgG isotypes^31,34^. Therefore, a human IgG4 isotype was selected for huPMN310 to achieve neutralization and clearance of AβO without causing inflammation.

In addition to an IgG4 isotype, the lack of binding to plaque and Aß vascular deposits observed with huPMN310 is also expected to contribute to a favorable safety profile (Fig. 3). In AD patients, antibodies that bind insoluble Aß aggregates, such as bapineuzumab and aducanumab, are associated with an increased risk of ARIA^17,20^. In contrast, solanezumab, which preferentially binds soluble monomers, has not caused ARIA in clinical trials^14^. The mechanism responsible for ARIA is believed to involve engagement of parenchymal and vascular Aß deposits causing the recruitment and activation of microglial cells, an effect mediated by an IgG1 effector isotype^18,19^. Indeed, administration of crenezumab, which binds to insoluble Aß aggregates but has an IgG4 isotype, resulted in only a low incidence (6 of 75 patients) of ARIA-H at high doses (30–60 mg/kg) with no cases of ARIA-E in a Phase 1b trial and one case of ARIA-E in a Phase 2 trial^25^. By comparison, patients dosed with aducanumab, which binds plaque and vascular aggregates and has an IgG1 effector isotype, experienced a high incidence of ARIA (41% at 10 mg/kg), significantly limiting the therapeutic window of the antibody in a Phase 1b study^20^. The selectivity of huPMN310 for soluble AßO, in conjunction with an IgG4 isotype, is expected to allow for the safe administration of high doses of the antibody, thereby maximizing the effective dose reaching the pathogenic target.

AßO are heterogeneous and examination of soluble Aß species in AD brain extracts by several investigators has indicated that the neurotoxic activity resides primarily in the LMW (<70 kDa) fraction of Aß oligomers as opposed to Aß monomers or HMW (>70 kDa) aggregates of Aß. Overall, analysis of soluble human AD brain extracts by various methodologies, including SEC, ultracentrifugation and immunoprecipitation, shows the presence of a range of different sized aggregates of Aß dominated by HMW species and less abundant smaller oligomeric species consisting of dimers, trimers, tetramers and dodecamers^16,35–37^. In a variety of assays, the LMW Aß oligomers, but not Aß monomers, exhibit potent neuronal toxicity causing neurite degeneration, disruption of cytoskeleton microtubules and decreased synaptic function *in vitro*, and memory impairment when injected into the brain of rodents *in vivo*^7–9,12,16^. By comparison, preparations of soluble HMW Aß aggregates from AD brains have been reported to be largely inert^16,36^, although evidence of *in vitro* toxicity was observed by some investigators^38^. Interestingly, it has been reported that HMW Aß aggregates can act as a reservoir for toxic oligomers by dissociating into LMW species^16^. The variability in results obtained with soluble HMW Aß species may be due to a number of factors including methodological differences in the preparation of brain fractions and potential overlap in the operational definition of LMW vs HMW.

The ability of huPMN310 to bind the LMW toxic oligomer-enriched fraction from AD brains was assessed in comparison to aducanumab and bapineuzumab (Fig. 6). In SPR assays, huPMN310 showed reproducibly high and preferential binding to the LMW vs HMW fraction of soluble AD brain extracts. The binding was confirmed to be specific for Aß through capture-detection and competition SPR assays. By comparison, aducanumab and bapineuzumab showed lower, non-preferential binding to the LMW fraction. These results demonstrate the greater selectivity of huPMN310 for native toxic AßO in AD brains.

In order to be efficacious, Aß-directed antibodies must cross the BBB and reach therapeutic levels in the CNS. In spite of the low CNS penetrance of circulating antibodies (0.1–1%), several lines of evidence indicate that these levels are sufficient to have an impact. For example, multiple autoimmune encephalitides are caused by circulating pathogenic antibodies entering the CNS (e.g. anti-NMDAR, anti-GABAR)^39^. Similarly, systemic delivery of antibodies or active immunization with Aß in animal models have been demonstrated to counteract pathological processes in the CNS^20,40,41^. Most importantly, clinical evidence has convincingly shown that systemic delivery of aducanumab results in the clearance of Aß plaque (and CNS side effects) measured by PET imaging^20^. The CNS penetrance of huPMN310 delivered systemically was evaluated in aged WT mice and was found to be comparable to that of aducanumab. A time course study conducted in aged transgenic APP/PS1 mice showed the presence of declining but detectable plasma and brain levels out to day 21, again similar to what has been reported for aducanumab^20^. These results suggest that huPMN310 can achieve CNS exposure comparable to that of aducanumab and other clinically tested monoclonal antibodies. Recent advances in strategies to enhance transport across the BBB could also be applied in the future to further improve CNS access.

In conclusion, our results support the potential of huPMN310 for improved efficacy due to its ability to selectively target pathogenic AßO without unproductive binding to non-toxic monomers and fibrils, as well as improved safety by virtue of its IgG4 isotype and lack of binding to plaque and vascular Aß deposits associated with ARIA. Therefore, the combination of selectivity and potential to safely administer high doses of antibody is expected to result in enhanced safety and therapeutic potency.

## Materials and Methods

### Peptide synthesis

Computational modeling using collective coordinates^22^ identified a conformationally- constrained Aβ primary sequence HHQK likely to be selectively exposed on oligomers while inaccessible to antibody binding in fibrils. The predicted conformational epitope was rarely present in the unstructured monomer ensemble. The conformational epitope was synthesized as a cyclic peptide with additional N-terminal residues CG and a C-terminal G to recapitulate the predicted structure of HHQK on AβO. Peptide synthesis was performed by CPC Scientific Inc. (Sunnyvale CA, USA) following standard manufacturing procedures. Peptide sequence was confirmed by electrospray mass spectral analysis. Purity was assessed by HPLC with a SepaxGP-C18 column and was determined to be 95%. Cyclization was performed via a head-to-tail (C-G) amide bond and c[CGHHQKG] was then conjugated to either keyhole limpet hemocyanin (KLH) or bovine serum albumin (BSA) via maleimide-based coupling. Non-cyclized, linear CGHHQKG peptide was also produced by CPC Scientific as a conformational negative control peptide.

### Immunization and ELISA screening of monoclonal antibodies

Immunization of mice for generation of monoclonal antibodies, and initial screening of hybridoma clones by enzyme-linked immunosorbent assay (ELISA) were performed by ImmunoPrecise Antibodies (Victoria BC, Canada) following established standard protocols^40^. Balb/c mice were immunized with cyclic peptide conjugated to KLH (c[CGHHQKG]-KLH), and splenic lymphocytes were collected on day 19 for hybridoma cell line generation.

Tissue culture supernatants from the hybridomas were screened by ELISA for reactivity to c[CGHHQKG]-BSA, and lack of reactivity to linear CGHHQKG-BSA or human transferrin as an irrelevant antigen^40,42^. Briefly, plates were coated overnight with 100 μl/well of 1 μg/ml c[CGHHQKG]-BSA or 1 μg/ml CGHHQKG-BSA in carbonate buffer (pH 9.6) at 4 °C, or 50 μl/well of 5 μg/ml human transferrin in distilled water (dH_2_O) at 37 °C. Hybridoma supernatant reactivity was detected by the addition of horseradish peroxidase (HRP)-conjugated goat anti-mouse IgG secondary antibody followed by the substrate 3,3′,5,5′- tetramehylbenzidine (TMB). Absorbance was read at 450 nM.

### Antibodies

The murine PMN310 (muPMN310) monoclonal antibody used in the studies presented was either purified from hybridoma supernatant by ImmunoPrecise Antibodies or produced by WuXi Biologics (Hong Kong, China) by transient transfection of Chinese hamster ovary (CHO) cells with a vector encoding the hybridoma variable regions cloned into a murine IgG1 framework. Humanized PMN310 (huPMN310) was generated by Abzena (Cambridge, UK) by cloning the humanized variable region genes of muPMN310 into vectors encoding a human IgG4 (S241P hinge variant) heavy chain constant domain and a human kappa light chain constant domain. Aducanumab and bapineuzumab were purchased from Creative Biolabs (Upton NY, USA). Negative isotype controls including murine IgG1, human IgG1 and human IgG4 were purchased from BioLegend (San Diego CA, USA).

### Aβ monomers and oligomers

Hexafluoroisopropanol (HFIP)-treated recombinant Aβ42 peptide (rPeptide, Watkinsville, GA, USA) was reconstituted in dimethyl sulfoxide (DMSO) (Sigma-Aldrich Canada, Oakville, ON) to give a stock concentration of 5 mM. To prepare monomers, the peptide solution was diluted further to 100 µM in dH_2_O and used immediately. Oligomers were prepared by diluting the peptide solution in phenol red-free F12 medium (Thermo Fisher, Waltham MA, USA) to a final concentration of 100 µM and incubated for 24 h at 4 °C followed by immediate use or storage at −80 °C.

### Brain extract

Brain tissues from 17 different human AD patients and 4 control individuals were obtained from brain banks affiliated with the University of British Columbia, the NIH NeuroBioBank and the National Prion Disease Pathology Surveillance Center (NPDPSC) at Case Western Reserve University. Donor characteristics are provided in Supplementary Table 1. Informed consent for tissue collection at autopsy and neurodegenerative research use was obtained from the legal representative in accordance with local institutional review boards. These studies were reviewed and approved by the UBC Ethics Board and are in accordance with the Declaration of Helsinki principles. The clinical diagnosis of AD was based on NINCDS-ADRDA criteria. Samples from frontal cortex were weighed and submersed in ice-cold Tris-buffered saline (TBS) (20% w/v) with EDTA-free protease inhibitor cocktail (Roche Diagnostics, Laval QC, Canada), and homogenized using an Omni tissue homogenizer (Omni International Inc, Keenesaw GA, USA), 3 × 30 sec pulses with 30 sec pauses in between, all performed on ice. Homogenates were then subjected to ultracentrifugation at 100,000 × g for 60 min. Supernatants (soluble extracts) were collected, aliquoted and stored at −80 °C. The protein concentration was determined using a bicinchoninic acid (BCA) protein assay. Pools of brain extracts from 3–8 patients were used in each analysis.

### Size exclusion chromatography

Pooled soluble brain extracts were injected at 0.5 ml/min through a Superdex 75 (10/300) HPLC column (GE Healthcare Life Sciences, Pittsburg PA, USA) for 50 min and 0.25 ml fractions were collected. Molecular weight (MW) markers (Bio-Rad Laboratories, Mississauga ON, Canada) were run separately. Protein peaks were monitored by absorbance at O.D. 280 nm. Fractions corresponding to a MW of ~8 kDa to ~70 kDa were pooled into a low molecular weight (LMW) fraction. Aβ monomers (MW ~4.5 kDa) were excluded from the LMW fraction. Fractions corresponding to a MW of >140 kDa to ~700 kDa were pooled into a high molecular weight (HMW) fraction. The LMW and HMW fractions were concentrated and total protein concentration was determined in a BCA assay. The fractions were then diluted to 100 μg/ml in phosphate-buffered saline, 3 mM EDTA, 0.05% surfactant P20 (PBS-EP) (GE Healthcare Life Sciences, Pittsburg PA, USA) containing BSA (2 mg/ml) for surface plasmon resonance (SPR) analysis.

### Surface plasmon resonance analysis

Surface plasmon resonance measurements were performed using a Molecular Affinity Screening System (Sierra Sensors, Hamburg, Germany). The cyclic structured peptide c[CGHHQKG] and the unstructured linear form of the peptide CGHHQKG were immobilized on high amine capacity (HAC) sensorchips (Sierra Sensors, Hamburg, Germany) and antibodies (15 µg/ml) were injected over the immobilized surfaces at 10 µl/minute for 4 min followed by a dissociation phase. In a separate set of experiments, antibodies were immobilized on the sensorchips and synthetic Aβ peptide monomers and Aβ oligomers were injected over the immobilized surfaces at 10 µl/min for 15 minutes. To assess the binding of antibodies to native AβO, SEC fractions of pooled soluble human AD brain extracts (100 µg/ml) were injected over the immobilized antibodies at 10 µl/min for 8 minutes. In SPR sandwich assays fractionated brain extracts were injected as above followed by a brief 1 minute dissociation and injection of detection antibody for 8 minutes. The binding responses from the resultant sensorgrams were double-referenced against unmodified reference surfaces and blank buffer injections. SPR results are expressed as binding response units (RU) to provide a quantitative assessment of overall antibody-ligand interactions in samples with multiple, heterogeneous species present at undefined concentrations such as in oligomer preparations and soluble brain extracts.

### Measurement of total Aβ and aggregated Aβ in brain extract

The amount of aggregated Aβ and total Aβ (monomers and aggregates) in the LMW and HMW fractions of pooled human AD soluble brain extract (pool from 3 brains) were measured at QPS (Grambach, Austria). Total amounts of Aβ38, 40 and 42 were determined by QPS in a commercial immunosorbent assay on a Meso Scale platform (MSD, Rockville MD, USA) using peptide standards. Aggregated Aβ levels were measured using the Amorfix Aggregated Aβ Assay (A4).

### Immunohistochemistry

Fresh frozen AD brain sections with no fixation were exposed to antigen retrieval citrate buffer (Target Retrieval Solution, Dako, Santa Clara CA, USA) for 20 min and incubated in a humidified chamber with serum-free protein blocking reagent (Dako) for 1 h to block non-specific staining. The sections were incubated overnight at 4 °C with primary antibodies (muPMN310, huPMN310, aducanumab, bapineuzumab, isotype controls) at 1 µg/ml and washed 3 times for 5 min in TBS containing 0.1% Triton-X-100 (TBS-T) buffer. Secondary HRP-conjugated rabbit anti-human IgG (0.4 μg/ml; Abcam, San Francisco CA, USA) or sheep anti-mouse IgG (1 μg/ml; GE Healthcare, Chicago IL, USA) antibodies were added to the sections and incubated for 1 h, followed by 3 washes in TBS-T buffer. Secondary antibody was also added to sections that were not exposed to primary antibody as a negative control. The HRP enzyme substrate, biaminobezidine (DAB) chromogen reagent (Vector Laboratories, Burlingame CA, USA), was then added to the sections followed by rinsing with dH_2_O. The sections were counterstained with haematoxylin QS (Vector Laboratories, Burlingame CA, USA). The slides were examined under a light microscope (Zeiss Axiovert 200 M, Carl Zeiss Toronto ON, Canada) and representative images were captured using a Leica DC300 digital camera and software (Leica Microsystems Canada Inc., Vaughan ON, Canada).

### *In vitro* propagation assay

Monomeric Aβ42 peptide was solubilized in 10 mM NaOH to a concentration of 500 μM, sonicated for 10 min, diluted to 50 μM in 1 mM EDTA Tris-HCL buffer (pH 7.4), and immediately added to a black-walled 96-well microtitre plate (Greiner Bio-One, Monroe NC, USA). Equal volumes of buffer alone, 10 μM muPMN310 test antibody or 10 μM irrelevant mouse IgG control were added to the wells, for a final volume of 100 μL and a 1:5 molar ratio of antibody:Aβ42 peptide. Thioflavin T (ThT) was added (10 μM final) and plates were incubated at room temperature for 24 h at 25 °C, with ThT fluorescence measurements (excitation at 440 nm, emission at 486 nm) recorded every hour using a Wallac Victor3v 1420 Multilabel Counter (PerkinElmer, Waltham MA, USA). Fluorescent readings were double-referenced by buffer alone and antibody only wells.

At end-point, samples were collected and the wells washed with 10 mM NaOH. The collected samples were centrifuged at 18,000 *g* for 14 min at 4 °C, and pellets were resuspended in 10 mM NaOH. After solubilization in sample buffer and boiling for 5 min at 95 °C, supernatants and pellets were run on a denaturing SDS-PAGE gel for monomerization and immunoblotting with a pan-Aβ antibody (6E10, BioLegend, San Diego CA, USA). Total Aβ in the pellet or supernatant appears as an approximately 4 kDa band.

### *In vitro* neurotoxicity assay

*In vitro* assessment of the ability of muPMN310 to inhibit the neurotoxicity of Aβ42O was performed by SynAging (Vandoeuvre-les-Nancy, France) following established protocols. Cortical neurons were prepared from embryonic day 16–17 C57Bl6/J mouse fetuses and cultured at 50,000 cells/well in 48-well plates pre-coated with 1.5 μg/ml polyornithine at 35 °C in a humidified 6% CO_2_ atmosphere. SynAging stable Aβ42O (consisting primarily of tetramers^43^, Supplementary Fig. 4) were pre-incubated for 30 min in the presence or absence of varying concentrations of muPMN310. Control wells with muPMN310 alone were included to test for any potential toxicity of the antibody itself. The final concentration of AβO was 1 μM and final concentrations of muPMN310 ranged from 0.1–2 μM. Neuronal cell cultures were then incubated with AβO and antibody (triplicate wells, 140 μL total volume) for 24 h after which cell viability was assessed in an MTT assay.

### *In vivo* neurotoxicity assay

*In vivo* assessment of the ability of muPMN310 to inhibit the neurotoxicity of AβO was performed by SynAging using behavioral and biochemical outcome measures. SynAging conducted animal experiments according to European guidelines for the care and use of laboratory animals, approved by the Direction Départementale de la Protection des Populations de Meurthe & Moselle – Domaine Expérimentation Animale and all protocols were submitted and approved by the local ethical committee. Groups of twelve C57Bl6/J mice (3 months of age) received an intracerebroventricular (i.c.v) injection of either AβO alone (50 pmoles), AβO plus muPMN310 (molar ratio of 1:2), muPMN310 alone, or vehicle alone. In all cases, a total volume of 5 μl was injected stereotactically into the right lateral ventricle using a 10 μl Hamilton microsyringe fitted with a 26-gauge needle and performed under anesthesia with ketamine/xylazine. Antibody and AβO were pre-incubated for 30 min prior to injection. Cognitive performance in a novel object recognition (NOR) test was performed on days 7–8. The experimenter was blinded to the treatment and all trials were video recorded (Smart v3.0 software, Bioseb) for quantification of time spent exploring the novel object. A discrimination index (DI) was generated: (DI) = (time exploring novel object − time exploring familiar object)/total exploration time. On day 10 post-treatment, brains were collected after intra-cardiac flash-perfusion with 0.9% saline under anesthesia. Hippocampi from individual mice were isolated, homogenized and exposed to 3 freeze-thaw cycles. The lysates were centrifuged at 800 × *g* for 15 min and the supernatants analyzed for concentrations of TNFα (pro-inflammatory marker), PSD-95 (postsynaptic marker) and SNAP25 (presynaptic marker) by SynAging using commercially available ELISA kits according to manufacturer’s recommendations (Cloud-Clone Corp., Houston TX, USA).

### Central nervous system exposure

APP/PS1 (APPswe/PSEN1dE9) and wild type C57BL/6 (WT) littermates were a gift from Dr. Cheryl Wellington, breeding pairs were originally purchased from Jackson Laboratories, Bar Harbor, ME. Animals were housed in accredited facilities in accordance with UBC and international animal ethics guidelines. The studies were reviewed and approved by the UBC Animal Care Committee.

Aged APP/PS1 and WT littermates (54–71 weeks old) were injected intraperitoneally (i.p.) with 30 mg/kg huPMN310, aducanumab or vehicle (PBS) as a negative control. Plasma was collected prior to sacrifice and on days 1,7,14 and 21 for assessment of circulating levels of human IgG. Animals were sacrificed immediately after plasma collection and PBS perfusion. Brains were then collected and flash frozen. Brains and plasma were stored at −80 °C until use. Brain homogenates (10% w/v) were generated in radioimmunoprecipitation assay (RIPA) buffer with an Omni tissue homogenizer (Omni International, Inc.), at half power for 30 sec, three times, with a 30 sec pause in between. Homogenates were then sonicated for 15 sec at half power, followed by clearance of debris by centrifugation at 2,000 × *g* for 10 min. Levels of human IgG in plasma (1:10,000 dilution) and brain homogenates (1:5 dilution) from individual mice were measured using the Human IgG Immunotek ELISA (Zeptomatrix, Buffalo NY, USA) according to manufacturer’s instructions.

### Statistical analysis

Statistical analysis was performed with GraphPad Prism 7 as described in the figure legends.

## Supplementary information

Supporting Information and Data

## Data Availability

The datasets generated during and/or analysed during the current study are available from the corresponding author on request. The monoclonal antibodies generated during and/or analysed during the current study are available under materials transfer agreement through the corresponding author.
