# Supplementary material for: A Rationally Designed Humanized Antibody Selective for Amyloid Beta Oligomers in Alzheimer’s Disease
Source: Sci Rep. 2019 Jul 8;9:9870. doi: 10.1038/s41598-019-46306-5 (PMC6614461; doi:10.1038/s41598-019-46306-5)
Supplement: Supplementary file 1 — Supporting Information and Data [file 41598_2019_46306_MOESM1_ESM.pdf]

Supplementary Material

A Rationally Designed Humanized Antibody Selective for Amyloid Beta Oligomers in Alzheimer's Disease

Ebrima Gibbs[1,+], Judith M. Silverman[1,+], Beibei Zhao[1], Xubiao Peng[2], Jing Wang[1], Cheryl L. Wellington[1], Ian R. Mackenzie[1], Steven S. Plotkin[3,4], Johanne M. Kaplan[4], Neil R. Cashman[1,4*]

[1]University of British Columbia, Djavad Mowafaghian Centre for Brain Health, Vancouver, BC, V6T 2B5, Canada
[2]University of British Columbia, Department of Physics and Astronomy, Vancouver, BC, V6T 1Z1, Canada
[3]University of British Columbia, Department of Physics and Astronomy and Genome Sciences and Technology Program, Vancouver, BC, V6T 1Z1, Canada
[4]ProMIS Neurosciences, Cambridge, MA, 02142, USA
*neil.cashman@vch.ca
[+]these authors contributed equally to this work

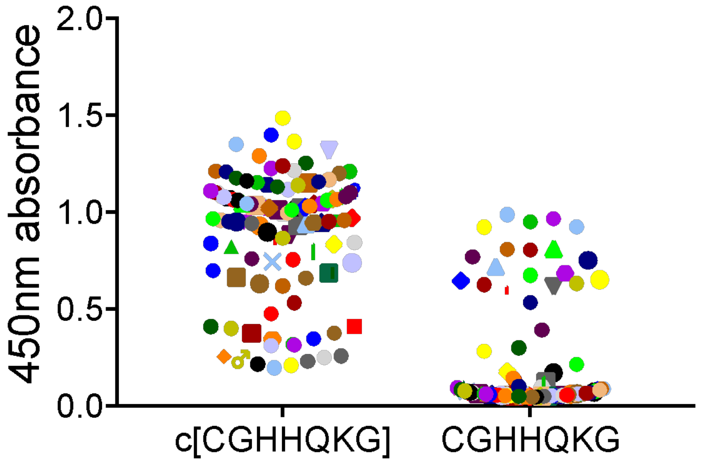

Supplementary Fig 1. ELISA screening of hybridoma clones generated by immunization with c[CGHHQKG]. Hybridoma supernatants from all clones were screened by ELISA. Clones with strong binding to the cognate conformational c[CGHHQKG] peptide and little or no reactivity to the linear unstructured CGHHQKG peptide were selected for further analysis.

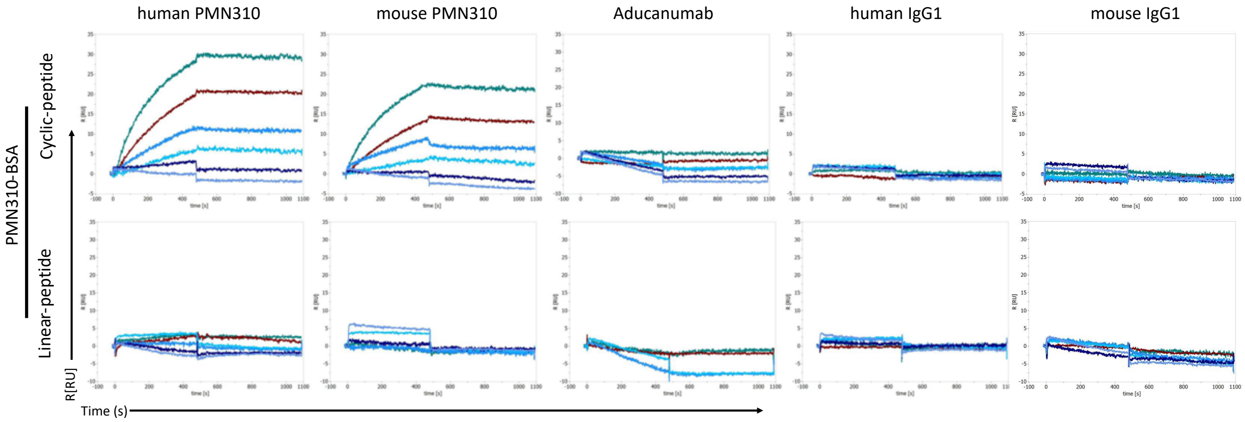

Cyclic peptide	Mouse PMN310	Humanized PMN310
**kA**	**7.514E+005 /(M·s)**	**9.141E+005 /(M·s)**
**kD**	**9.081E-005 /s**	**3.287E-005 /s**
**K_D**	**1.209E-010 M**	**3.596E-011 M**
**X^2**	**0.1434 RU^2**	**0.08834 RU^2**

Supplementary Fig 2. High affinity binding of humanized and mouse PMN310 to cyclic peptide epitope vs linear peptide. 6-Point, 2-Fold serial dilutions (5nM to 0.156nm) analysis of BSA-conjugated cyclic, c[CGHHQKG], and linear, unstructured CGHHQKG peptide binding to indicated antibodies was performed by SPR. Sensorgrams are representative of at least 3 identical experiments.

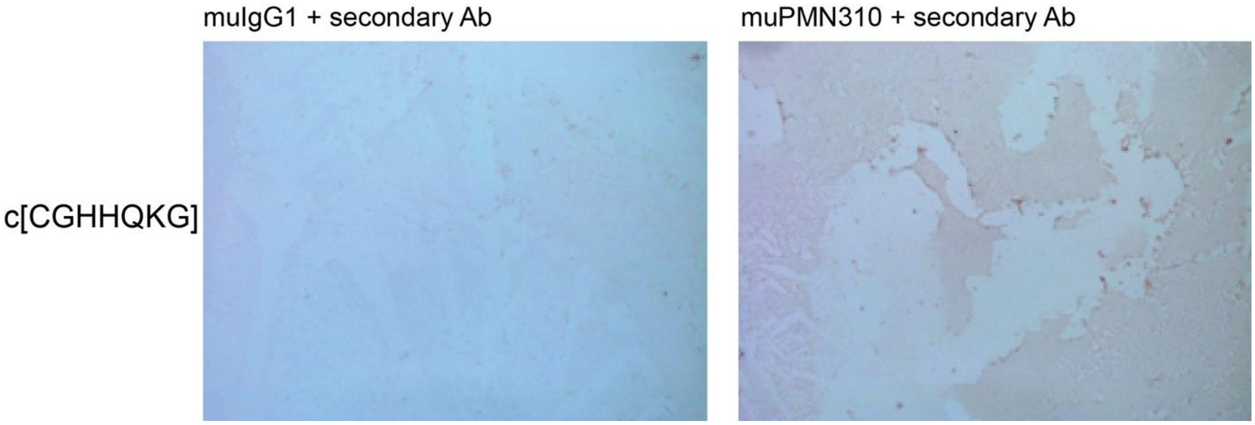

Supplementary Fig 3. Conformational epitope of PMN310 is detectable under staining conditions. To verify that the lack of staining observed with PMN310 antibodies was not due to alteration of its conformational epitope under the staining conditions used, BSA-conjugated c[CGHHQKG] peptide was dried on a slide that was then submitted to the same staining protocol as the brain tissue sections. Positive brown staining was observed on slides stained with muPMN310 and not control muIgG1 indicating that the conformational epitope was indeed recognized by muPMN310 under the staining conditions used. Ab = antibody.

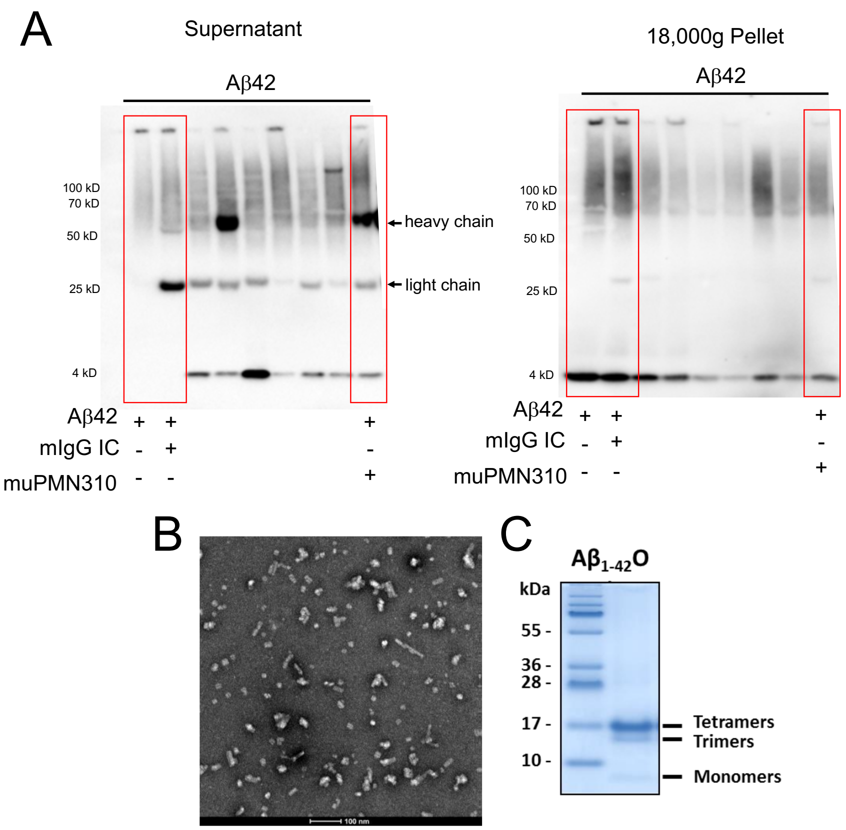

Supplemental Fig 4. A) Complete uncropped immunoblots from Fig. 4b. Beta-sheet formation was tracked for 24 hours in vitro by a Thioflavin-T fluorescent assay after addition of Aβ monomers alone, or in the presence of a non-specific IgG1 isotype control (both in left red box) or PMN310 (right red box) at a molar ratio of 1:5 (Antibody:Aβ). Middle lanes represent screening of other monoclonal antibodies. Samples were collected at assay end point and fractionated into soluble (Supernatant) or insoluble (Pellet) Aβ by centrifugation, then resolved on SDS-PAGE for immunoblotting with pan-Aβ antibody, 6E10. The heavy and light chains of the mouse monoclonal antibodies used in the assay are detected by the anti-mouse-HRP conjugated detection antibody used in immunoblotting. Shown are representative data from one of two identical experiments. **B-C) Characterization of stable Amyloid beta (1-42) oligomers.** B) Electron micrograph of negatively stained stable AβO from SynAging. C) Coomassie stained SDS-PAGE of SynAging AβO. AβO was separated by 15 % SDS-PAGE gels under non-reducing condition and stained with Coomassie blue. The oligomeric preparation contains a mixture of stable dimers, trimers and tetramers of $A\beta_{1-42}$, as well as few remaining monomeric forms of the peptides. This coomassie stained gel image adheres to the digital image and integrity policies of Scientific Reports. Reproduced with permission from: T. Pillot., N. Fischer, P. Housset, Y. Terroire, S. Lemoine, A. Allouche, P. Goetghebeur, A. Köpke, V. Koziel. (2017, September). Prion-like soluble misfolded protein oligomers induce neurodegeneration: disease models. Poster session presented at 47th European Brain and Behaviour Society Meeting, BILBAO (Spain).

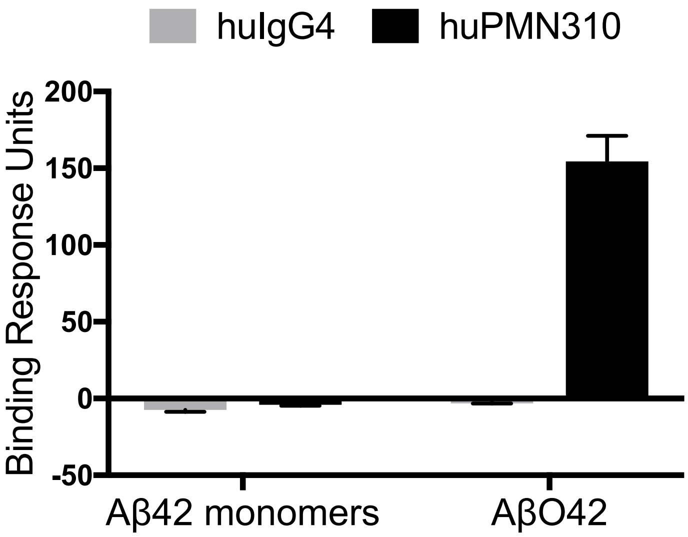

Supplementary Fig 5. Humanized PMN310 retains selectivity for Aβ oligomers. The binding of humanized PMN310 (huPMN310) to Aβ42 monomers and oligomers (AβO42) was assessed by SPR. The humanized version of the muPMN310 monoclonal antibody retained preferential recognition of oligomers vs monomers. N=3, Error bars = SEM

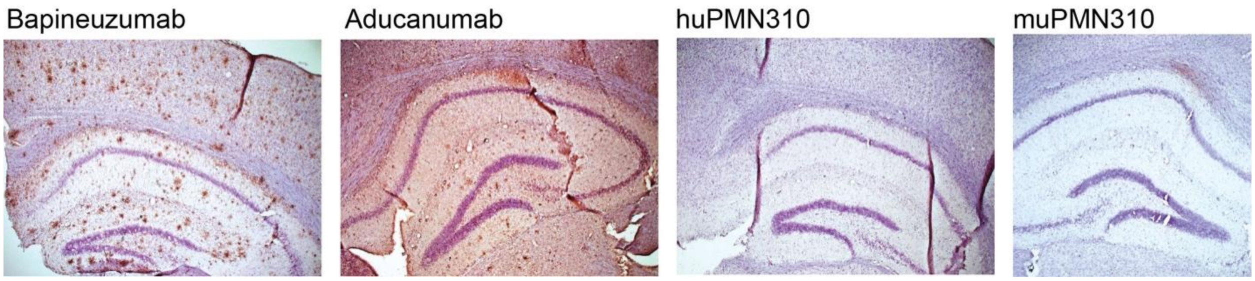

Supplementary Fig 6. PMN310 does not react with Aβ plaque in brain sections from APP/PS1 transgenic mice. Frozen brain sections from aged APP/PS1 transgenic mice were stained with the indicated antibodies. Detection of bound antibody with secondary anti-human or anti-mouse IgG appears in brown, and nuclear counterstaining with hematoxylin in blue. Neither muPMN310 nor huPMN310 showed any staining above background while bapineuzumab and aducanumab showed robust plaque staining.

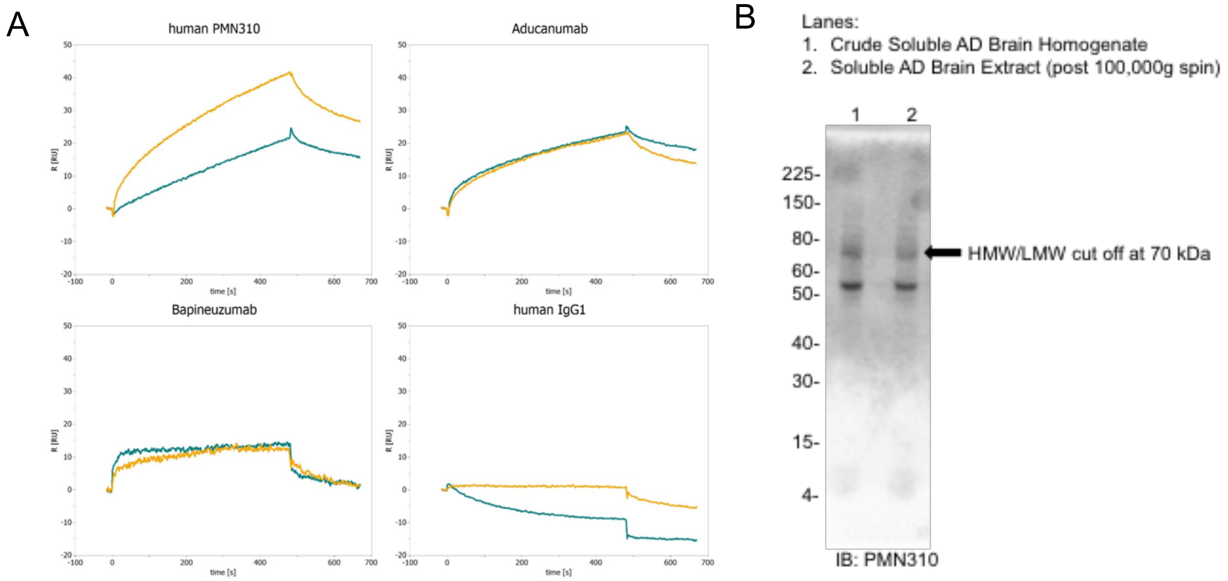

Supplementary Fig 7. huPMN310 preferentially recognizes the toxic oligomer-enriched LMW fraction of pooled soluble AD brain extract. A) Representative SPR sensorgrams of pooled (n=4) AD brain extract LMW (yellow) and HMW (green) fractions binding to the indicated antibodies. B) muPMN310 direct immunoblotting of AD brain crude homogenate (Lane 1) and subsequent brain extract (Lane 2) after fractionation by SDS-PAGE. Location of Molecular Weight Markers are indicated on the left, arrow indicates the LMW/HMW cut-off (70 kDa) used in the SPR experiment (panel A).

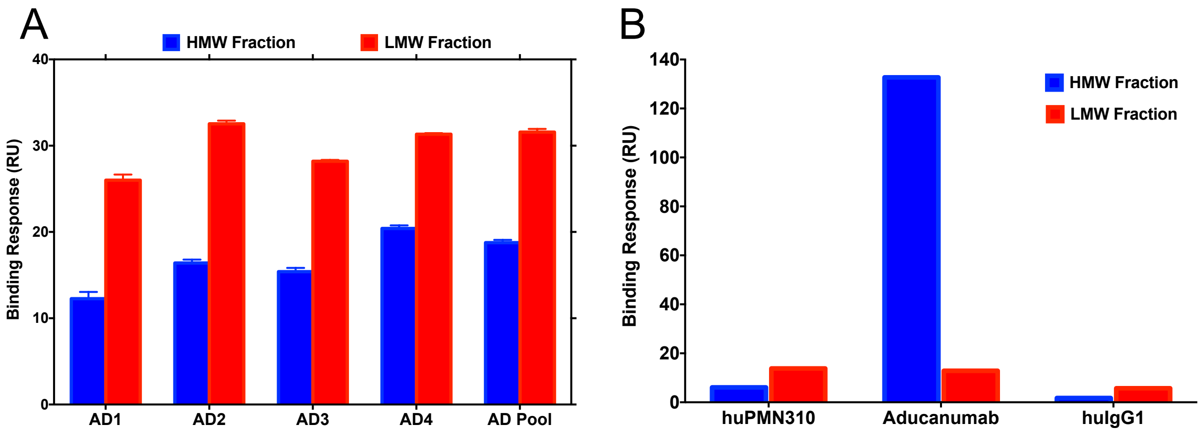

Supplementary Fig 8. Preferential binding of huPMN310 to the LMW fraction of soluble brain extracts. A) Binding of huPMN310 to LMW and HMW fractions from four individual AD brains, and a pool of those brains assessed by SPR. B) Binding of huPMN310 and aducanumab to LMW and HMW fractions from four pooled non-AD brain extracts assessed by SPR.

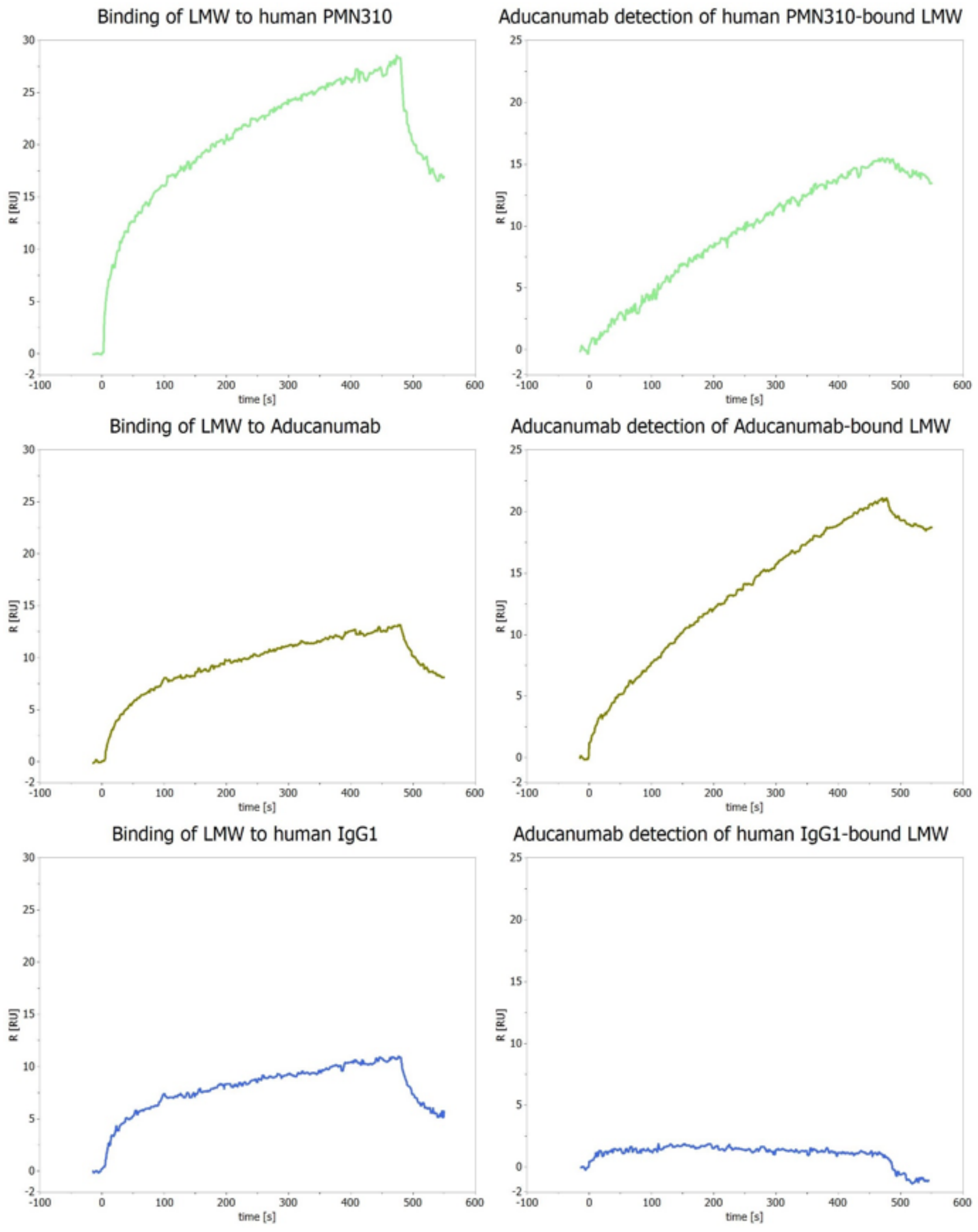

Supplementary Fig 9. Representative SPR sensorgrams of LMW AD brain extract binding to indicated antibodies and subsequent aducanumab detection of the captured analyte.

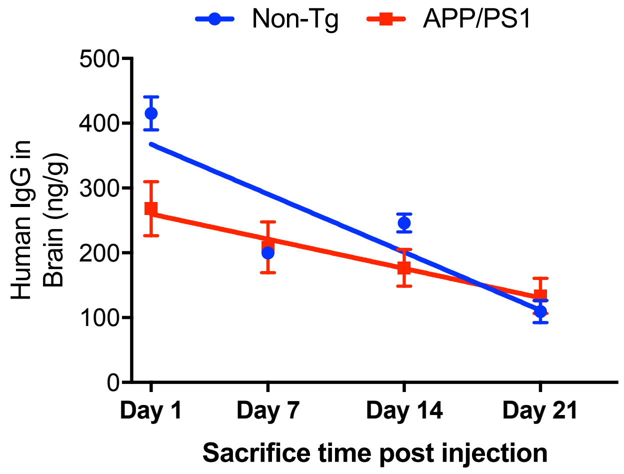

Supplementary Fig 10. Trend for greater retention of huPMN310 in APP/PS1 mice vs WT mice. Linear regression analysis of the slopes for brain levels of huPMN310 over time shows a relatively slower decline in APP/PS1 mice (slope: -6.46 $\pm$ 0.73) compared to WT mice (slope: -12.81 $\pm$ 5.28) but the difference does not reach statistical significance ($p = 0.299$).

Supplementary Table 1

Sample ID Number	Sex	Age	PMI	Control vs. AD
777	M	22	4	Control
1793	M	11	19	Control
1037	M	19	11	Control
1226	M	23	21	Control
1172	F	79	12	AD
1630	F	84	5	AD
1312	F	87	7	AD
RES-01	F	77	26	AD
RES-02	M	59	24	AD
RES-03	M	56	48	AD
RES-04	F	80	135	AD
RES-05	F	56	35	AD
RES-06	M	71	28	AD
RES-07	M	75	n/a	AD
RES-08	F	71	55	AD
NDBB 029	M	73	<24 hrs	AD
NDBB 084	M	77	<24 hrs	AD
NDBB 167	F	76	<36 hrs	AD
NDBB 170	M	62	n/a	AD
NDBB 197	F	72	<36 hrs	AD
NDBB 093	F	81	<48 hrs	AD

Table 1. Donor characteristics of brain samples used in the studies.
n/a = Not available, PMI = Post-mortem interval, AD = Alzheimer's disease
